# Communicating Health to Young Adults Using Social Media: How, Where, and When?

**DOI:** 10.3390/nu14142967

**Published:** 2022-07-20

**Authors:** Clare F. Dix, Linda Brennan, Tracy A. McCaffrey, Mike Reid, Annika Molenaar, Amy Barklamb, Shinyi Chin, Helen Truby

**Affiliations:** 1 School of Human Movement and Nutrition Sciences, The University of Queensland, Brisbane 4076, Australia; h.truby@uq.edu.au; 2 School of Media and Communication, RMIT University, Melbourne 3004, Australia; linda.brennan@rmit.edu.au (L.B.); shinyi.chin@rmit.edu.au (S.C.); 3 Department of Nutrition, Dietetics and Food, Monash University, Notting Hill 3168, Australia; tracy.mccaffrey@monash.edu (T.A.M.); annika.molenaar@monash.edu (A.M.); amy.barklamb@monash.edu (A.B.); 4 School of Economics, Finance and Marketing, RMIT University, Melbourne 3000, Australia; mike.reid@rmit.edu.au

**Keywords:** communication, health, social marketing, young adults, health promotion, social media

## Abstract

Communication with young adults about healthy lifestyle behaviours needs to result in improvements in dietary choices to impact the prevalence of diet-related diseases. This paper presents the health beliefs, behaviours, and communication practices in young Australian adults (*n* = 2019) by their pre-defined psycho-behavioural characteristics: Lifestyle Mavens, Health-Conscious, Aspirational Healthy Eaters, Balanced-All Rounders, Contemplating Another Day, or Blissfully Unconcerned. The Lifestyle Mavens and Health-Conscious groups were more likely to actively seek out health information on social media (*p* < 0.05). Lifestyle Mavens were the most likely to engage with health and food content on social media, whereas the Blissfully Unconcerned were the least likely to engage (*p* < 0.05). Lifestyle Mavens are more likely to report creating food and health-related content for social media, whereas Aspirational Healthy Eaters are more likely to report searching for food and health-related content online, but are less likely to share or create content. Contemplating Another Day are more likely to report interactions with commercial content. This paper defines how psycho-behavioural segments communicate about health, where they look for information, how they may prefer to receive health messages, and when they are most receptive to messages. By applying existing robust market segmentation techniques, this paper provides nuanced information that challenges the assumption that online social media health information is preferred by all young adults.

## 1. Introduction

Young adults have been the target of many health promotion campaigns but are proving a ‘hard to reach’ group in terms of adoption of healthy behaviours, as evidenced by 18–24-year-olds having a rapid weight trajectory [1]. Young Australians (18–24 years) have the lowest consumption of fruit and vegetables compared to older adults so, if we are to stem the rise of obesity in this age group, the need for effective communication about prevention of weight gain is clear [2].

In their scoping review, Munt et al. [3] identify barriers and enablers of healthy eating in young adults. They describe key barriers to healthy eating as: lack of time to shop, prepare and cook healthy foods, perceptions that unhealthy foods are cheaper than healthy options, and peer pressure in conforming to norms. These are all connected with consuming unhealthy foods, as are prevailing expectations of social groups to consume certain foods in social situations [3].

In 2021, 90% of young adults were engaging regularly with social media, such as Facebook, Instagram, YouTube, and Twitter [4,5]. This provides opportunities to both be exposed to, and create, health information and experiences [4]. Social marketing and the use of social media offer significant opportunities for health behaviour change, integrated campaigns and customised messages for specific young-adult target market segments in a way that has not previously been available to health and public health practitioners [6]. The use of social media facilitates proactive two-way and many-to-many communication, in contrast to passive one-way traditional media such as TV, radio, and print [5]. This provides both challenges and opportunities as it enables rapid dissemination of misleading information as well as evidence-based messages [7]. Social media has the potential to increase the reach and engagement (browsing, liking, commenting, and sharing content) with health-related messaging given the strong theoretical foundations [8]. However, there is a dearth of studies that demonstrate the actual success of using social media to communicate health messages to young adults, which points to the fact that consumer engagement may be inadequately operationalised. Understanding the needs of young adults as a target audience and measuring the quality of their engagement and retention in the social media campaigns will provide a deeper dimension to the meaning of ‘success’ [9].

In the existing literature of young adults, stratification by a single variable such as ‘sex’ is commonly described; for example, in Munt’s et al [3] review it is reported that males have a greater apathy towards making healthy dietary choices compared to females [3]. This knowledge would perhaps prompt an intervention aimed at young men; for example, to eat more fruit and vegetables. However, targeting a campaign only by a grouping variables such as demographic, location of work or study, and/or a single physiological variable (such as sex) overlooks the subtle differences that exist within these groups which are critical for adopting the desired behaviour, that is attitudes, beliefs and intentions [10], as well as the behavioural ecosystem in which these behaviours occur [11]. To address this gap, we have recently applied marketing techniques to define six distinct market segments or “Living and Eating for Health Segments” (LEHS) with the specific purpose of these segments or personas being a mechanism to stratify young adults into pre-defined groups based on their psycho—behavioural characteristics, rather than by physiological variables or demographics [12]. This research, “Communicating Health”, aims to bridge the gap between nutritionist, media, and social marketing professionals to produce tools that can be used to improve engagement with young adults and reduce the prevalence of obesity. Further analysis provided a deep understanding of psycho-behavioural characteristics of specific segments which can be applied for campaign design [13]. This paper extends this work and explores health information seeking behaviours within each specific segment, what platforms they are most likely to be using, and when they are actively seeking information about health. This knowledge can be translated into practice by health promotion experts when operationalising campaigns, enabling approaches matched to their similarities in behavioural intentions, beliefs, and preferences for communication routes.

## 2. Materials and Methods

This study arises from multi-disciplinary research, “Communicating Health”, in which the purpose was to develop and test the tools that may be used to improve engagement with young Australian adults using social media. Ethical approval for the study has been provided by Monash University Human Research Ethics Committee (approval number 17629) and informed consent was obtained from all participants. The full methodologies are described in the study protocol [14]. In brief, the research aimed to identify psycho-behavioural characteristics of young adults in Australia. Development and validation of the LEHS involved a four-stage mixed method design. 

### 2.1. LEHS Development

The design and validation of the six psycho-behavioural LEHS utilized a four-stage mixed method design [15] and was underpinned by the Integrated Model of Behaviour Change [16]. See Table 1 for the persona descriptors of the LEHS. These phases incorporated the findings of the literature reviews, and qualitative and quantitative data. Three literature reviews provided context on the food and health environment online for young adults [17,18,19], including the impact of social media on body image and nutrition, and Indigenous Australian perspectives on the impact of social media on nutrition. Qualitative data were gathered from 195 young adults via online conversations on health and well-being with a focus on food and the role of social media in shaping decisions about food and eating. Participants completed 20 forums, 2 online challenges, 3 short polls, and journal entries through facilitated discussions over a 4-week period [20,21,22,23]. Preliminary profiles were then developed using a multi-stage process with a multi-disciplinary research team. These preliminary profiles, or LEHS, were assessed and defined quantitatively via an online survey (*n* = 2019). Participants were recruited by an Australian Market and Social Research Society-certified field house (Qualtrics ®), and detailed methodology can be found in the study protocol [12]. Participant eligibility criteria included being between the ages of 18 and 24 years and currently residing in Australia. Quotas were set to achieve an Australian nationally representative sample for gender (48% female, 47% Male, and 5% other) and location metro (67.3%) and regional/remote (32.7%) based on the 2016 Australian Census [24]. The survey consisted of 46 closed-ended questions along with self-reported height and weight [25] (https://doi.org/10.26180/5dba10f4ec6e5) (accessed on 18 January 2022). Questions gathered information on:

- Demographics
- Causes of obesity (adapted from Allison et al. [26] and Ata et al. [27])
- Quality of life (adapted from Rocha et al. [28] and Meiselman [29])
- Nutrition knowledge (adapted from Mitchison et al. [30] and Flynn et al. [31])
- Body image satisfaction (adapted from Mitchison et al. [30])
- Food and food preparation skills (adapted from McGowan et al. [32])
- Meal skipping (adapted from Kutsuma et al. [33])
- Normative beliefs, motivations (adapted from Fishbein and Ajzen [34])
- Attitudes towards food and eating (adapted from Naughton et al. [35]).
- Intention to seek health-related information [36]
- Their self-perceived nutrition knowledge [31,37]
- If, where, and how they sought health information from different online sources (adapted from *Sensis Social Media Report* 2017 [38])
- How they engaged with health information (passive or active) (adapted from *Digital Behaviors Segmentation* [39])
- Their behavioural intentions towards the health information they retrieved (Adapted from Siuki [40])

In this paper, we present the results on the intention to seek health-related information [36], self-perceived nutritional knowledge [31,37], health information seeking behaviours (adapted from *Sensis Social Media Report 2017* [38]), health information engagement (adapted from *Digital Behaviors Segmentation* [39]), and behavioural intentions towards sought health information (adapted from Siuki [40]). Supplementary Table S1 provides detailed explanation of the questions and possible answers posed in relation to these topics.

In previous articles, we described the detailed methodology of developing the six different psycho-behavioural LEHS profiles or market segments for young adults; defined as Lifestyle Mavens, Health-Conscious, Aspirational Healthy Eaters, Balanced-All Rounders, Contemplating Another Day, and Blissfully Unconcerned [12,13,14].

### 2.2. Statisticial Analysis

Individual questions were grouped together by similar characteristics for analyses (see Supplementary Table S1). Statistical analyses were conducted using IBM SPSS statistics^®^ version 25 (Armonk, NY, USA). Differences between psychographic and other lifestyle and behavioural variables assessed in the survey were evaluated using One-way ANOVA with post-hoc testing. Significance was set at *p* < 0.05 except where a Bonferroni correction was applied to pairwise comparisons.

## 3. Results

### 3.1. Demographics

This sample of young adults has been characterized by detailed demographics published previously [13]. A total of 2019 young adults aged from 18 to 24 years old, residing in Australia, completed the online survey in December 2018, with a mean age of 21 (SD 2) years, n = 906 (44.9% females), n = 1046 (51.8% males), n = 62 non-binary/gender fluid/gender queer/ trans-gender (3.1%) and n = 5 (0.2%) preferred not to assign themselves a gender. This sample was categorized into one of the six LEHS; Lifestyle Mavens (n = 311, 15.4%); Health-Conscious (n = 425, 21.1%), Aspirational Healthy Eaters (n = 556, 27.5%), Balanced All-Rounders (n = 432, 21.4%), Contemplating Another Day (n = 26, 11.2%) and Blissfully Unconcerned (n = 69, 3.4%). Sample participants were drawn from diverse cultural backgrounds, although the majority in each LEHS identified as Oceanic (Australian, New Zealander, or Polynesian). Very few (1.3%) had not completed Year 9 education and the Lifestyle Mavens (11.9%) were most likely to have completed post-graduate study. Living arrangements were diverse with no between group differences, with the majority living in family households with or without non-family members present, 9.8% living alone, and 13% living in shared households with non-family members. Weight and height were self-reported, and n = 1274 (63%) would be classified by BMI as under a healthy weight and n = 745 (37%) would be classified above a healthy weight (BMI >25kg/m^2^) (See Supplementary Table S2).

### 3.2. Health Information Seeking Behaviours and Social Media

In examining where young adults are likely to seek health information, there were significant differences between segments (Table 2). Lifestyle Mavens and Health-Conscious individuals were more likely to access health resources from government sources (*p* < 0.05) and more likely to access health resources from health and wellness blogs and forums, product reviews, and advertisements (*p* < 0.05) than other LEHS. Those individuals who were classified as Contemplating Another Day, Balanced All-Rounders, or the Blissfully Unconcerned were the least likely to access health resources from health and wellness blogs and forums, product reviews, and advertisements (*p* < 0.05). Lifestyle Mavens and Health-Conscious individuals were more likely to actively seek out health information on social media (*p* < 0.05), which was not a source of health information for those Contemplating Another Day, Balanced All-Rounders, or the Blissfully Unconcerned (*p* < 0.05). Lifestyle Mavens, Health-Conscious, and Aspirational Healthy Eaters had the greatest intention to search online for health information, compared to Contemplating Another Day and Blissfully Unconcerned individuals (*p* < 0.05). 

**Figure 1:**
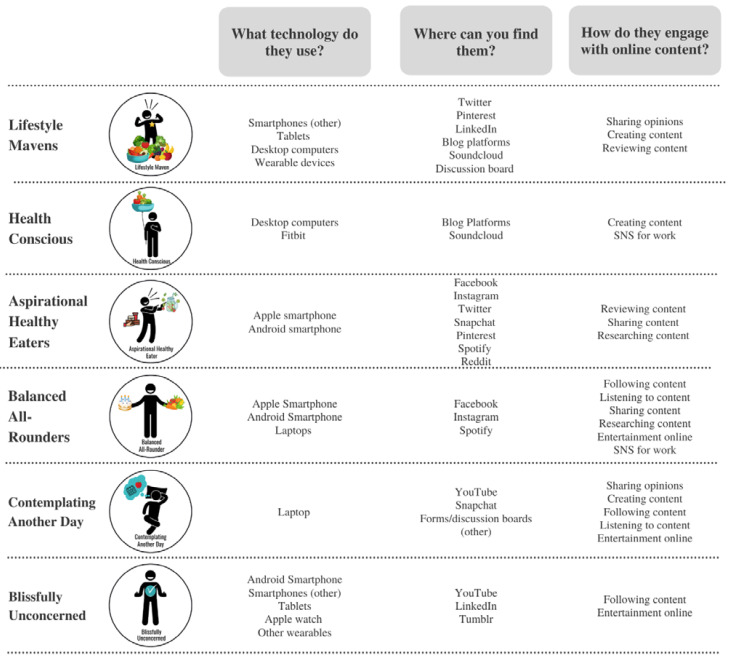
Summary of online behaviours most likely to be extensively seen in young adults defined by Living and Eating for Health Segments (LEHS). See Supplementary Tables S3−S5 for survey response percentages.

Figure 1 depicts the various social media platforms that different LEHS frequent with key differences in platform preferences between groups, what technology they are using to support their social media usage, and how they engage with online content. Lifestyle Mavens report the most engagement with social media and use the broadest range of technological platforms, followed by Aspirational Healthy Eaters who are more likely to report sharing opinions and creating and reviewing content. Individuals who are designated as Health-Conscious are more likely to use desktop computers and Fitbits, and report highest use of Soundcloud and blog platforms. They are more likely to create online content and use social media for work. Aspirational Healthy Eaters are more likely to use smartphones, and report high use of Facebook, Instagram, Twitter, Snapchat, Pinterest, and Spotify. They are more likely to report reviewing, sharing, or researching content online. Balanced All-Rounders are more likely to use smartphones and laptops, and report high use of Facebook, Instagram, and Spotify. These individuals report being more likely to follow, listen to, and research online content, engage with online entertainment, and use social media for work. Individuals who are designated as Contemplating Another Day are most likely to use a laptop. They report high use of YouTube and Snapchat, and share, create, follow, or listen to content, and engage with online entertainment. Blissfully Unconcerned individuals are more likely to use smartphones, tablets, and wearable devices, and to report high use of YouTube and Tumblr. They are more likely to follow content online or engage with online entertainment. 

**Figure 2:**
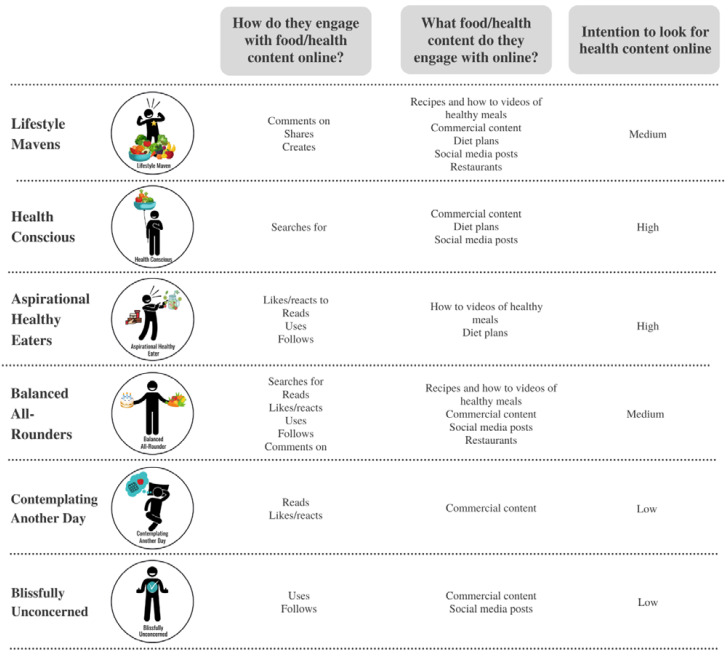
Summary of food and health-related online behaviours most likely to be extensively seen in young adults defined by Living and Eating for Health Segments (LEHS). See Table 2 and Table 3 for survey response percentages.

Figure 2 details differences between LEHS in food and health-related content, specifically, their intention to look for food or health-related content, what types of content they are most likely to look for, and how they engage with the content. In terms of how often health information was purposely sought and engaged with via social media, differences between groups were apparent. The Aspirational Healthy Eaters were the most likely to report searching, reading, reacting to, and using online information on healthy food, diet plans, commercial content, social media posts from individuals and restaurants. The Blissfully Unconcerned reported the least engagement with all online content on health food, diet plans, commercial content, social media posts, and restaurants. Active engagement such as commenting and sharing social media content was different between segments (Table 3). Lifestyle Mavens were most likely to report commenting on healthy food recipes, commercial content, and diet plans, and were the most likely to share privately or publicly healthy food recipes, diet plans, commercial content, and social media content from restaurants or from individuals. Lifestyle Mavens were also significantly more likely than other groups to create healthy food recipes, how to videos, commercial content, and diet plans. Those defined as Aspirational Healthy Eaters were the most likely to comment on others’ (individuals) social media posts, and to share healthy food recipes privately and publicly. Aspirational Healthy Eaters were also the group most likely to use all types of content, followed by Balanced All-rounders, and Health-Conscious individuals. Health-Conscious individuals were the most likely to publicly share health food recipes and comment on healthy food recipe videos and social media posts by restaurants. Those Contemplating Another Day and Blissfully Unconcerned were less actively engaged on social media and were the least likely segments to engage with any food-related content. 

## 4. Discussion

This paper describes young adults’ food and health-related information seeking behaviours and intentions using social media. As such, it provides a nuanced understanding of their habitual use, preferences, and likely engagement with health information sources provided via social media, and taken in the context of the earlier papers [12,13], provides additional direction for those wishing to create health-promotion social media campaigns. Reported engagement with social media components of interventions varies widely from 3 to 69% [17]. These data paint a picture of the diverse nature of young adults’ online health information seeking behaviours, as well as illustrating how likely they are to engage with content. This information can be used to inform health promotion campaigns using social media.

An important finding from this research is that young adults who share similar demographic characteristics differ widely when it comes to online health information seeking behaviours. Currently, health promotion strategies employing social media design campaigns with broad reach and appeal miss the mark when it comes to specific sub-groups. Commonly, demographic and geographic variables are used to segment populations, and although useful for intervention design, these criteria do not account for the behaviour of populations and its influence on campaign engagement [41]. Psychographic segmentation provides a greater understanding of individual motivators for actions and behaviours [12,42]; for example, the demographics of Health-Conscious and Aspirational Healthy Eaters are very similar, but their approach to accessing and engaging with online content is different. Health-Conscious individuals are more likely to create content on a computer and Aspirational Healthy Eaters more likely to use a smartphone to share or review content. 

The integration of traditional segmentation strategies with psycho-behavioural information can provide a more effective approach to intervention development [43]. Previous food and nutrition research that utilize psycho-behavioural segmentation has consistently used post-hoc segmentation methods, where segments are determined after data has been collected [44]. This prevents interventions being tailored to individual segments behaviours, as these are unknown. Our results suggest that health promotion agencies seeking to establish behaviour change are unlikely to affect many young adults using this type of communication and campaign strategy. For example, a campaign designed to support advocacy for change could engage with the Lifestyle Mavens but could result in reactance and push back from the Blissfully Unconcerned. Whereas a priori determination of psycho-behavioural segments allows for the tailoring of campaign strategies to each persona, increasing engagement and uptake, and maximising resource effectiveness. 

The use of social media has been widely touted as being a panacea for reaching young adults who are not actively engaged in traditional media. However, our results indicate that the groups most in need of assistance using social media for health are using it the least. For example, the Blissfully Unconcerned, in addition to not being concerned about their health, also actively avoid information about health propagated via social media. They do not actively search for information, and they are the least likely to use veracious sources of information overall. The Blissfully Unconcerned are also less likely to be attracted to content developed by others, even their close social networks. Although only marginally interested (3%) overall, they are interested in commercial content (5.3%) only when such content is provided by their close social network (5.5%). Consequently, affecting the behaviours of the Blissfully Unconcerned with social media is unlikely and will take more systemic interventions or changes [45]. Environmental changes that promote and provide access to healthier food and drink options, combined with the use of segmentation to design health interventions, have the potential to influence these less motivated individuals. 

In addition to identifying different groups, these results illustrate the need for nuanced consideration of psycho-behavioural variables in campaign design. Each of the LEHS identified differs in important ways from the other groups in terms of their behaviours in what they search for, read, and engage with. This indicates that reaching a particular group will require a different campaign strategy. For example, Aspirational Healthy Eaters are likely to search for and respond to recipes for healthy food, as well as diet plans. However, Lifestyle Mavens are most likely to advocate and support healthy lifestyles, and do not seek out that information and are, therefore, less likely to pass it on to those in need of the information. In these circumstances content creation will be an important strategy for reaching this group. The search–read–react ratio provides some interesting insight into the behaviour of different groups. Young adults will read things that they were not searching for, which bodes well for online campaign construction. However, their reactions (liking and sharing) are more muted and varied between LEHS, and they clearly do not react to all they see on social media. Consequently, campaign effectiveness metrics that measure only ‘likes’ and ‘shares’ are unlikely to capture the whole picture and tracing behaviour changes to social media campaigns will remain problematic. 

Social media engagement is conversational and iterative, not monologic and didactic, which can often be the approach of traditional health promotion campaigns. The use of co-design and co-creation in developing online health promotion campaigns for young adults could address this issue [46]. Co-design includes a range of stakeholders, including end users, in the design process. Co-creation involves several individuals participating in collaborative creation and has been shown to improve engagement with social media content. This approach would allow young adults to participate and actively contribute to the communication developed in an intervention or campaign [46]. Furthermore, targeted recruitment of specific LEHS of interest in this process would allow bespoke interventions to be developed increasing reach and effectiveness to those with similar attitudes and beliefs. For example, encouraging users to create content (Lifestyle Mavens and Health-Conscious individuals) could lead to sharing and reviewing of content by Aspirational Healthy Eaters and Balanced All-rounders.

The value of these findings perhaps lies not in who social media health campaigns are reaching but who they are not. Clearly, Lifestyle Mavens and Health-Conscious individuals are more likely to actively seek out health information on social media and share it with others, extending its reach. However, these groups are already attuned to healthy living. The groups that health promoters need to engage with are those who are Contemplating Another Day, Balanced All-rounders, or the Blissfully Unconcerned. Each of these groups has specific issues with how they engage with social media for health, and consequently, a one-size-fits-all strategy is unlikely to succeed. This analysis demonstrates that using social media platforms to affect behaviour change for these groups may not be useful. Health professionals in the online health space may need to take alternative approaches. For example, engagement with groups who are most likely to publicly share on their networks (Lifestyle Mavens) and using existing influencers to connect with at risk groups [47]. Indeed, social media may not be the communication method of choice for connecting and influencing those Blissfully Unconcerned at all and connecting to such individuals may require very different approaches. 

The understanding of which groups share health information widely and thereby influence others is also an important finding from this body of work. As it is not only evidence-based information that is shared, social media also rapidly disseminates misinformation. Here we examine which sources of information are ‘trusted’ [48], that is, are viewed as a source of truth and, therefore, such messages from trusted sources are more likely to be adopted into actual behaviours. Lifestyle Mavens and the Health-Conscious were more likely to seek information from government and other reputable sources which is an important and reassuring finding. Being able to harness these segments to propagate, by sharing publicly and privately, provides an avenue for evidence-based information to be disseminated. However, these sources of truth are not high on the information seeking priorities of other young adult segments. Health promoting agencies need to understand who is accessing their information and who is unlikely to be touched by their web presence. This information again supports the need to further understand how to effectively engage with under-served groups who are currently not touched by current social media presence.

Strengths of this research include the use of a large and varied group of young adults who were recruited from across Australia by a commercial marketing company. Limitations include the fact that this is a cross-sectional analysis and that social media platforms move and change fluidly. The nature of the sample precludes the ability to analyse social media channel use and development or loss of trust attributable to the COVID-19 pandemic and we acknowledge that motivations to change behaviour may be impacted by the pandemic. 

In summary, this analysis provides information to health promoters in how and why young adults use social media to search and engage with health information on social media. The nuanced approach using market segments suggests that social media is one communication channel for health information but is not the panacea to reach all young adults. 

## Figures and Tables

**Table 1:** Living and Eating for Health Segments (LEHS) persona descriptors.

Lifestyle Mavens	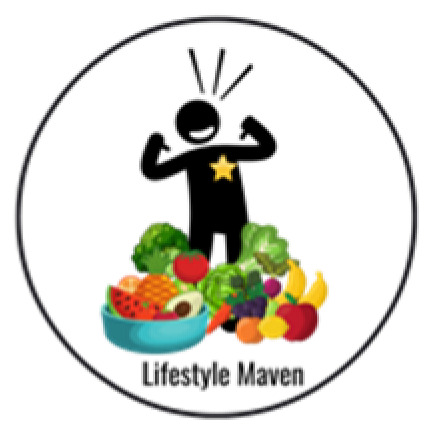	I am passionate about healthy eating and health plays a big part in my life. I use social media to follow active lifestyle personalities or get new recipes/exercise ideas. I may even buy superfoods or follow a particular type of diet. I like to think I am super healthy.
Health Conscious	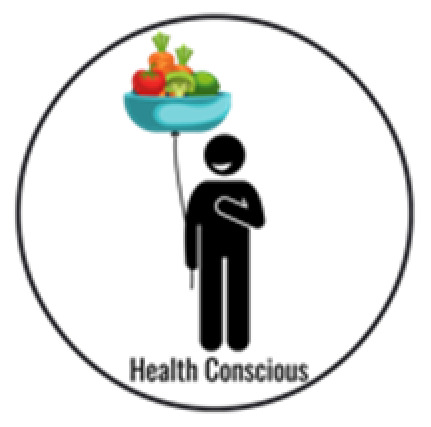	I am health-conscious and being healthy and eating healthy is important to me. Although health means different things to different people, I make conscious lifestyle decisions about eating based on what I believe healthy means. I look for new recipes and healthy eating information on social media.
Aspirational Healthy Eaters	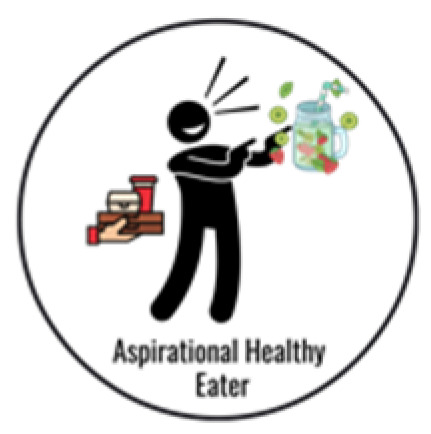	I aspire to be healthy (but struggle sometimes). Healthy eating is hard work! I have tried to improve my diet, but always find things that make it difficult to stick with the changes. Sometimes I notice recipe ideas or healthy eating hacks, and if it seems easy enough, I will give it a go.
Balanced All Rounders	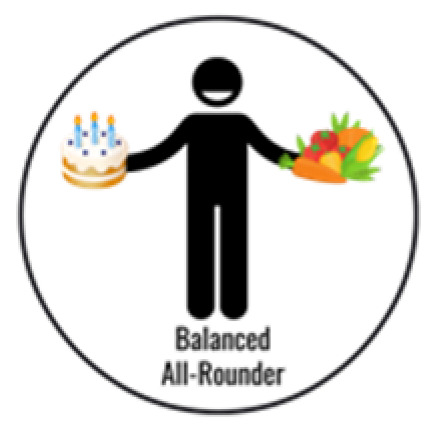	I try and live a balanced lifestyle, and I think that all foods are okay in moderation. I should not have to feel guilty about eating a piece of cake now and again. I get all sorts of inspiration from social media like finding out about new restaurants, fun recipes, and sometimes healthy eating tips.
Contemplating Another Day	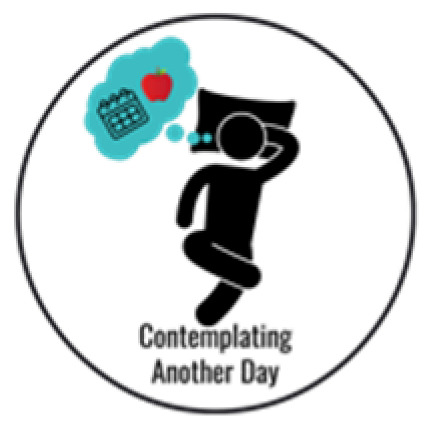	I am contemplating healthy eating, but it is not a priority for me right now. I know the basics about what it means to be healthy, but it does not seem relevant to me right now. I have taken a few steps to be healthier, but I am not motivated to make it a high priority because I have too many other things going on in my life.
Blissfully Unconcerned	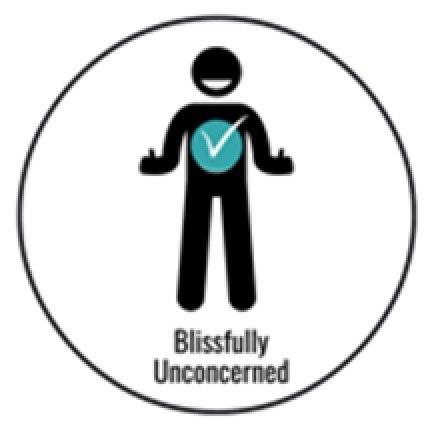	I am not bothered about healthy eating. I do not really see the point and I do not think about it. I do not really notice healthy eating tips or recipes and I do not care what I eat.

**Table 2:** Health information seeking behaviours by Living and Eating for Health Segments (LEHS) (n = 2019).

Characteristic	Category	Lifestyle Mavens *n =* 311	Health Conscious *n =* 425	Aspirational Healthy Eaters *n =* 556	Balanced All-Rounders *n =* 432	Contemplating Another Day *n =* 226	Blissfully Unconcerned *n =* 69
Seeking Online Health Resources	WHO, WebMD, Mayo, Government, State Gov health websites	10.9 (3.87) ^a^	10.1 (4.01) ^a^	8.6 (4.08) ^b^	8.3 (3.90) ^b^	7.8 (3.83) ^b^	8.2 (3.85) ^b^
	Health and wellness blogs, Health and medical forums, reviews of medical health products, ads of medical health products	11.9 (4.18) ^a^	10.3 (4.32) ^b^	8.5 (4.06) ^c^	7.7 (3.79) ^c,d^	7.4 (4.09) ^d^	8.5 (4.34) ^c,d^
	Friends and family posts, other people with similar health concerns on social media, YouTube wellness channels	9.1 (2.86) ^a^	8.3 (3.15) ^b^	7.5 (3.17) ^c^	7.1 (3.23) ^c,d^	6.7 (3.33) ^d^	7.1 (3.16) ^c,d^
Intention to search online for food/health information		9.9 (2.43) ^a,b^	10.1 (2.67) ^a^	10.1 (2.65) ^a^	9.5 (2.92) ^b^	8.1 (3.18) ^c^	8.0 (3.2) ^c^

Values in the same row and sub-table not sharing the same superscript are significantly different at *p* < 0.05 in the two-sided test of equality for column means. Cells with no superscript are not included in the test. Tests assume equal variances. Tests are adjusted for all pairwise comparisons within a row of each innermost sub-table using the Bonferroni correction.

**Table 3:** Social media usage and engagement by Living and Eating for Health Segments (LEHS) (*n =* 2019).

Action	Content	Lifestyle Mavens *n* = 311	Health Conscious *n* = 425	Aspirational Healthy Eaters *n* = 556	Balanced All-rounders *n =* 432	Contemplating Another Day *n =* 226	Blissfully Unconcerned *n =* 69
Search for	recipes of healthy food	12.8%	19.7%	30.2%	25.9%	9.9%	1.4%
	how to videos for healthy meals	10.4%	22.9%	30.1%	25.7%	9.6%	1.3%
	commercial content	13.9%	24.5%	25.2%	22.3%	11.0%	3.2%
	diet plans	12.1%	25.3%	30.2%	24.0%	7.2%	1.1%
	posts from family and friends about food/health	14.9%	24.0%	27.4%	21.9%	10.3%	1.5%
	restaurants	9.1%	21.2%	27.2%	28.8%	12.5%	1.2%
Read	recipes of healthy food	11.3%	20.5%	29.1%	26.1%	10.9%	2.1%
	how to videos for healthy meals	11.3%	22.4%	28.6%	27.1%	8.9%	1.7%
	commercial content	11.4%	20.0%	27.8%	25.3%	13.5%	1.9%
	diet plans	10.9%	23.2%	32.8%	22.1%	9.7%	1.3%
	posts from family and friends about food/health	9.2%	22.1%	30.6%	24.7%	11.8%	1.6%
	restaurants	10.3%	19.6%	29.2%	27.2%	12.0%	1.8%
Like/react to	recipes of healthy food	13.8%	19.5%	29.6%	23.5%	11.5%	2.1%
	how to videos for healthy meals	11.8%	20.6%	31.2%	24.0%	11.0%	1.4%
	commercial content	17.8%	20.4%	24.3%	21.4%	13.2%	3.0%
	diet plans	17.8%	22.3%	34.1%	18.6%	6.1%	1.1%
	posts from family and friends about food/health	11.3%	19.0%	30.3%	25.9%	12.0%	1.5%
	restaurants	14.4%	22.9%	24.6%	24.1%	11.9%	2.0%
Comment on	recipes of healthy food	28.4%	24.8%	22.0%	14.2%	7.1%	3.5%
	how to videos for healthy meals	26.2%	29.0%	24.8%	11.7%	5.6%	2.8%
	commercial content	31.1%	23.2%	23.2%	13.0%	7.3%	2.3%
	diet plans	28.2%	27.6%	23.8%	10.5%	8.3%	1.7%
	posts from family and friends about food/health	15.0%	18.5%	29.7%	23.9%	11.0%	1.9%
	restaurants	23.7%	25.3%	24.1%	15.6%	10.5%	0.8%
Share privately	recipes of healthy food	17.0%	22.9%	28.4%	19.0%	9.8%	2.9%
	how to videos for healthy meals	23.6%	21.2%	23.6%	20.4%	8.4%	2.8%
	commercial content	27.9%	23.9%	22.1%	13.7%	9.3%	3.1%
	diet plans	34.2%	17.8%	22.8%	14.6%	7.3%	3.2%
	posts from family and friends about food/health	23.4%	21.6%	25.5%	16.5%	9.4%	3.6%
	restaurants	21.9%	20.1%	24.2%	20.8%	10.0%	3.0%
Share publicly	recipes of healthy food	24.3%	26.3%	24.7%	18.1%	6.2%	0.4%
	how to videos for healthy meals	26.9%	20.8%	27.4%	15.6%	6.6%	2.8%
	commercial content	35.2%	21.4%	24.0%	9.7%	5.1%	4.6%
	diet plans	31.9%	25.8%	22.5%	12.1%	3.3%	4.4%
	posts from family and friends about food/health	30.0%	23.6%	24.1%	13.2%	7.3%	1.8%
	restaurants	26.3%	21.1%	23.7%	15.5%	10.3%	3.1%
Create	recipes of healthy food	31.1%	28.0%	20.1%	13.4%	5.5%	1.8%
	how to videos for healthy meals	33.1%	26.2%	20.0%	9.2%	8.5%	3.1%
	commercial content	36.8%	23.2%	18.1%	10.3%	9.0%	2.6%
	diet plans	36.2%	24.8%	17.0%	9.9%	9.2%	2.8%
	posts from family and friends about food/health	31.9%	23.3%	20.9%	10.4%	8.0%	5.5%
	restaurants	31.6%	23.5%	23.5%	11.8%	5.1%	4.4%
Use/ Follow	recipes of healthy food	10.0%	21.3%	30.9%	26.2%	10.5%	1.2%
	how to videos for healthy meals	11.2%	22.7%	27.6%	25.6%	11.2%	1.7%
	commercial content	16.1%	22.7%	29.9%	19.4%	6.6%	5.2%
	diet plans	14.2%	24.0%	34.2%	18.2%	7.4%	2.2%
	posts from family and friends about food/health	17.4%	21.8%	25.9%	21.8%	10.9%	2.0%
	restaurants	14.7%	18.7%	27.1%	25.8%	13.0%	0.7%

## Data Availability

The data are not publicly available due to privacy and ethical considerations as participants did not consent for their information to be made accessible on a public repository. A copy of the survey questions is available at https://doi.org/10.26180/5dba10f4ec6e5.

## Supplementary Materials

The following supporting information can be downloaded at https://www.mdpi.com/article/10.3390/nu14142967/s1, Table S1: Characteristics of measures as reported in Table 1. (https://doi.org/10.26180/5dba10f4ec6e5); Table S2: Characteristics, demographics, and self-reported weight of Living and Eating Health Segments (LEHS); Table S3: Technology usage by Living and Eating for Health Segments (LEHS); Table S4: Social media platform usage by Living and Eating for Health Segments (LEHS); Table S5: Online behaviours with social media content by Living and Eating for Health Segments (LEHS).
