# Supplementary material for: Communicating Health to Young Adults Using Social Media: How, Where, and When?
Source: Nutrients. 2022 Jul 20;14(14):2967. doi: 10.3390/nu14142967 (PMC9318771; doi:10.3390/nu14142967)
Supplement: Supplementary file 1 [file nutrients-14-02967-s001.zip › nutrients-1699518-supplementary.pdf]

Supplementary Table S1: Characteristics of measures as reported in Table 1. (https://doi.org/10.26180/5dba10f4ec6e5)

Characteristic	Question	Scoring Criteria	Maximum Score	Items
Perceived nutrition knowledge/expertise [31,37]	How much do you agree with the following statements?	5-point Likert Scale<br>1 – strongly disagree<br>5 – strongly agree	20	I know quite a bit about healthy eating<br>I do not feel very knowledgeable about healthy eating<br>When it comes to healthy eating, I really don't know a lot<br>Compared to most people, I know less about healthy eating
Nutrition knowledge Mavenism	How much do you agree with the following statements?	5-point Likert Scale<br>1 – strongly disagree<br>5 – strongly agree	10	In my circle of friends, I am one of the "experts" on healthy eating<br>People seek me out for information on healthy eating
Seeking Online Health Resources [40]	How often do you seek health information from these online sources?	5-point Likert Scale<br>1 – Rarely<br>5 – Usually	20	World Health Organisation website<br>WebMD, Mayo Clinic or similar medical health sites<br>Australian Government Department of Health (www.health.gov.au)<br>State Government Health Websites (e.g., Better Health Channel)
	How often do you seek health information from these online sources?	5-point Likert Scale<br>1 – Rarely<br>5 – Usually	20	Health and wellness blogs (e.g., https://jessicasepel.com/)<br>Health and medical forums (e.g., http://ourhealth.org.au)<br>Reviews of medical or health products<br>Advertisements of health or medical products and services
	How often do you seek health information from these online sources?	5-point Likert Scale<br>1 – Rarely<br>5 – Usually	20	Other people who have similar health concerns on social media (e.g., Facebook, Instagram)<br>Friends and Family posts on social media (e.g., Facebook, Instagram)<br>YouTube wellness channels<br>I intend to search for health-related information online over the next month
Intention to search online for food/health information (adapted from [36]	How much do you agree/disagree with the following statements about your intention to search for health and food information online?	5-point Likert Scale<br>1 – strongly disagree<br>5 – strongly agree	15	I intend to search for health-related information online over the next month<br>I intend to search for healthy eating and food related information online over the next month<br>I intend to search for healthy cooking recipes and meal plans online over the next month

Supplementary Table S2. Characteristics, demographics, and self-reported weight of Living and Eating Health Segments (LEHS).

Characteristic	Category	Lifestyle Mavens *n* = 311 (15.4%)	Health Conscious *n* = 425 (21.1%)	Aspirational Healthy Eaters *n* = 556 (27.5%)	Balanced All -Rounders *n* = 432 (21.4%)	Contemplating Another Day *n* = 226 (11.2%)	Blissfully Unconcerned *n* = 69 (3.4%)
Age (years)		21 (2)	21 (2)	21 (2)	21 (2)	21 (2)	21 (2)
Ethnicity	Oceanian	207 (66.6%)	290 (68.2%)	433 (77.9%)	322 (74.5%)	170 (75.2%)	48 (69.6%)
	Aboriginal or Torres Strait Islander	11 (3.5%)	15 (3.5%)	17 (3.1%)	13 (3.0%)	11 (4.9%)	6 (8.7%)
	North-West European	19 (6.1%)	27 (6.4%)	42 (7.6%)	42 (9.7%)	9 (4.0%)	2 (2.9%)
	Southern and Eastern European	15 (4.8%)	22 (5.2%)	16 (2.9%)	22 (5.1%)	10 (4.4%)	1 (1.4%)
	North African and Middle Eastern	12 (3.9%)	14 (3.3%)	6 (1.1%)	10 (2.3%)	5 (2.2%)	0
	South-East Asian	7 (2.3%)	23 (5.4%)	27 (4.9%)	37 (8.6%)	14 (6.2%)	3 (4.3%)
	North-East Asian	24 (7.7%)	43 (10.1%)	25 (4.5%)	30 (6.9%)	9 (4.0%)	4 (5.8%)
	Southern and Central Asian	28 (9%)	21 (4.9%)	35 (6.3%)	23 (5.3%)	15 (6.6%)	3 (4.3%)
	Peoples of the Americas	13 (4.2%)	9 (2.1%)	11 (2%)	8 (1.9%)	4 (1.8%)	1 (1.4%)
	Sub-Saharan African	8 (3.2%)	6 (1.9%)	1 (0.2%)	4 (0.9%)	3 (1.3%)	0
	Ethnicity Not Provided	10 (3.2%)	3 (0.7%)	10 (1.8%)	6 (1.4%)	3 (1.3%)	6 (8.7%)
Education Level	Never attended school	0	3 (0.7%) [a]	1 (0.2%) [a]	0	0	0
	Year 8 or below	2 (0.6%) [a]	2 (0.5%) [a]	5 (0.9%) [a]	3 (0.7%) [a]	4 (1.8%) [a]	1 (1.4%) [a]
	Year 9 or equivalent	3 (1.0%) [a,b]	1 (0.2%) [a]	0	2 (0.5%) [a]	1 (0.4%) [a,b]	3[b] (4.3%)
	Year 10 or equivalent	8 (2.6%) [a,b]	12 (2.8%) [a,b]	13 (2.3%) [a]	19 (4.4%) [a,b,c]	16 (7.1%) [b,c]	9c (13.0%)
	Year 11 or equivalent	10 (3.2%) [a]	17 (4.0%) [a]	26 (4.7%) [a]	23 (5.3%) [a]	15 (6.6%) [a]	6 (8.7%) [a]
	Year 12 or equivalent	92 (11.3%) [a]	164 (38.6%) [a,b]	255 (45.9%) [b]	191 (44.2%) [b,c]	93 (41.2%) [a,b]	22 (31.9%) [a,b]
	Certificate (non-high school)	6 (1.9%) [a,b]	5 (1.2%) [a,b]	7 (1.3%) [a,b]	4 (0.9%) [a]	2 (0.9%) [a,b]	4 (5.8%) [b]
	Certificate I/II (non-high school)	6 (1.9%) [a,b]	8 (1.9%) [a]	26 (4.7%) [a,b]	17 (3.9%) [a,b]	9 (4.0%) [a,b]	6 (8.7%) [b]
	Certificate III/IV (non-high school)	29 (9.3%) [a]	40 (9.4%) [a]	48 (8.6%) [a]	49 (11.3%) [a]	27 (11.9%) [a]	1 (1.4%) [a]
	Advanced diploma/diploma	19 (6.1%) [a]	34 (8.0%) [a]	41 (7.4%) [a]	23 (5.3%) [a]	23 (10.2%) [a]	3 (4.3%) [a]
	Bachelor's degree	75 (18.4%) [a]	109 (26.7%) [a]	107 (19.2%) [a,b]	78 (18.1%) [a,b]	29 (12.8%) [b]	10 (14.5%) [a,b]

Characteristic	Category	Lifestyle Mavens *n* = 311 (15.4%)	Health Conscious *n* = 425 (21.1%)	Aspirational Healthy Eaters *n* = 556 (27.5%)	Balanced All -Rounders *n* = 432 (21.4%)	Contemplating Another Day *n* = 226 (11.2%)	Blissfully Unconcerned *n* = 69 (3.4%)
	Graduate diploma/ graduate certificate	18 (5.8%)	12 (2.8%)	13 (2.3%)	8 (1.9%)	5 (2.2%)	2 (2.9%)
	Postgraduate degree	37 (11.9%) [a]	13 (3.1%) [b]	11[b] (2.0%) [b]	10 (2.3%) [b]	2 (0.9%) [b]	2 (2.9%) [a,b]
	Prefer not to say	6 (1.9%)	5 (1.2%)	3 (0.5%)	5 (1.2%)	0	0
Weekly Income (AUD$)	No income	24 (7.7%) [a]	57 (13.4%) [a,b]	59 (10.6%) [a,c]	40 (18.1%) [b,c]	40 (17.7%) [b,c]	11 (15.9%) [a,b]
	$1–$399	89 (28.6%)	114 (26.8%)	176 (31.7%)	71 (30.3%)	71 (31.4%)	30 (43.5%)
	$400–$649	39 (12.5%)	66 (15.5%)	96 (17.3%)	28 (13.9%)	28 (12.4%)	7 (10.1%)
	$650–$999	54 (17.4%)	59 (13.9%)	90 (16.2%)	34 (15.7%)	34 (15.0%)	6 (8.7%)
	$1000–$1499	46 (14.8%) [a]	63 (14.8%) [a]	46 (8.3%) [b]	23 (10.0%) [a,b]	23 (10.2%) [a,b]	7 (10.1%) [a,b]
	$1500–over $3000	47 (15.1%) [a]	45 (10.6%) [a,b]	47 (8.5%) [b,c]	14 (4.4%) [b,c,d]	14 (6.2%) [b,c,d]	3 (4.3%) [a,b,c]
	Prefer not to say	12 (9.3%)	21 (4.9%)	42 (7.6%)	16 (7.6%)	16 (7.1%)	5 (7.2%)
Living Arrangements	One family household with only family members present	142 (45.7%)	192 (21.2%)	252 (45.3%)	192 (44.4%)	97 (42.9%)	29 (42.0%)
	Two family household with only family members present	16 (5.1%)	24 (5.6%)	30 (5.4%)	23 (5.3%)	11 (4.9%)	2 (2.9%)
	Three or more family household with only family members present	21 (6.6%)	54 (12.7%)	65 (11.7%)	51 (11.8%)	28 (12.4%)	4 (5.8%)
	One family household with non-family members present	6 (1.9%)	18 (4.2%)	20 (3.6%)	20 (4.8%)	8 (3.5%)	1 (1.4%)
	Two family household with non-family members present	9 (2.9%)	5 (1.2%)	19 (3.4%)	10 (2.3%)	7 (3.1%)	0
	Three or more family household with non-family members present	18 (5.8%)	17 (4.0%)	21 (3.8%)	14 (3.2%)	6 (2.7%)	3 (4.3%)
	Lone person household	38 (12.2%)	44 (10.4%)	39 (7.0%)	40 (9.3%)	25 (11.1%)	13 (18.8%)
	Group household	36 (11.6%)	50 (11.8%)	75 (13.5%)	62 (14.4%)	31 (13.7%)	10 (14.5%)
	Prefer not to say	25 (8.0%)	21 (4.9%)	35 (6.3%)	20 (4.6%)	13 (5.8%)	7 (10.0%)
Studying Status	Studying full-time	111 (35.7%)	151 (35.5%)	191 (34.4%)	170 (39.4%)	95 (42%)	13 (18.8%)
	Studying part-time	60 (19.3%)	86 (20.2%)	106 (19.1%)	68 (15.7%)	36 (15.9%)	13 (18.8%)
	Not studying	122 (39.2%)	173 (21.1%)	233 (41.9%)	174 (40.3%)	82 (36.3%)	36 (52.2%)
	Prefer not to say	11 (3.5%)	8 (1.9%)	11 (2.0%)	9 (2.1%)	8 (3.5%)	6 (8.7%)

Characteristic	Category	Lifestyle Mavens *n* = 311 (15.4%)	Health Conscious *n* = 425 (21.1%)	Aspirational Healthy Eaters *n* = 556 (27.5%)	Balanced All -Rounders *n* = 432 (21.4%)	Contemplating Another Day *n* = 226 (11.2%)	Blissfully Unconcerned *n* = 69 (3.4%)
Working Status	Working full-time	144 (46.3%)	141 (33.2%)	140 (25.2%)	95 (22.0%)	39 (17.3%)	13 (18.8%)
	Working part-time	75 (24.1%)	111 (26.1%)	155 (27.9%)	97 (22.5%)	53 (23.5%)	13 (18.8%)
	Working casually	33 (10.6%)	64 (15.1%)	102 (18.3%)	92 (21.3%)	39 (17.3%)	12 (17.4%)
	Not working	50 (16.1%)	101 (23.8%)	146 (26.3%)	138 (31.9%)	90 (39.8%)	26 (37.7%)
	Prefer not to say	8 (2.6%)	5 (1.2%)	12 (2.2%)	8 (1.9%)	4 (1.8%)	5 (7.2%)
Body Mass Index (kg/m^2)		24.58 ^{a,d,e} (5.93)	23.40 (4.86) ^{a}	26.04 (6.66) ^{c}	23.73 (4.94) ^{a,b}	25.39 (6.32) ^{c,d}	26.27 (7.34) ^{b,c,e}
BMI Categories	Underweight (BMI < 18.5)	28 (9.0%)	42 (9.9%)	37 (6.7%)	41 (9.5%)	16 (7.1%)	9 (13.0%)
	Healthy weight (BMI 18.5–24.9)	171 (55.0%)	275 (64.7%)	260 (46.8%)	254 (58.8%)	111 (49.1%)	30 (43.5%)
	Overweight (BMI 25.0–29.9)	72 (23.2%)	76 (17.9%)	145 (26.1%)	87 (20.1%)	53 (23.5%)	13 (18.8%)
	Obese (BMI >30.0)	40 (12.9%)	32 (7.5%)	114 (20.5%)	50 (11.6%)	46 (20.4%)	17 (24.6%)

Values in the same row and sub-table not sharing the same subscript are significantly different at $p < 0.05$ in the two-sided test of equality for column means. Cells with no subscript are not included in the test. Tests assume equal variances. Tests are adjusted for all pairwise comparisons within a row of each innermost sub-table using the Bonferroni correction.

Supplementary Table S3: Technology usage by Living and Eating for Health Segments (LEHS) (n = 2019).

Device	Usage	Lifestyle Mavens *n* = 311	Health Conscious *n* = 425	Aspirational Healthy Eaters *n* = 556	Balanced All-rounders *n* = 432	Contemplating Another Day *n* = 226	Blissfully Unconcerned *n* = 69
Smartphone (Apple)	Do not use	13.8%^{a}	22.6%^{b}	27.3%^{bc}	33.1%^{c}	35%^{cd}	40.6%^{ce}
	Limited use	8.7%^{a}	4.7%^{ab}	4.1%^{ab}	3%^{b}	5.3%^{ab}	4.3%^{ab}
	2	5.8%^{a}	3.5%^{ab}	1.8%^{b}	2.3%^{ab}	4%^{ab}	0.0%
	3	18.3%^{a}	12%^{ac}	6.3%^{bd}	3.2%^{b}	5.8%^{bcd}	15.9%^{ad}
	4	19.9%^{a}	15.1%^{ab}	12.2%^{b}	10.9%^{bc}	10.2%^{bd}	7.2%^{ab}
	Extensive use	32.5%^{a}	41.9%^{ab}	48%^{b}	47.2%^{bc}	39.8%^{ab}	31.9%^{ab}
Smartphone (Android)	Do not use	26.7%^{a}	40%^{bd}	47.8%^{bc}	52.3%^{c}	46.5%^{bc}	24.6%^{ad}
	Limited use	8.7%^{a}	7.5%^{a}	7.2%^{a}	4.6%^{a}	4.9%^{a}	7.2%^{a}
	2	10.6%^{a}	6.6%^{ab}	4.1%^{abc}	1.9%^{bc}	3.1%^{bcd}	1.4%^{abc}
	3	17%^{a}	12.5%^{a}	6.1%^{b}	4.9%^{b}	9.3%^{ab}	20.3%^{a}
	4	16.7%^{a}	13.2%^{a}	6.3%^{b}	6.7%^{b}	8.8%^{ab}	8.7%^{ab}
	Extensive use	19%^{a}	20%^{a}	28.2%^{b}	29.4%^{b}	27.4%^{ab}	37.7%^{b}
Smartphone (Other)	Do not use	38.3%^{a}	62.1%^{b}	74.1%^{ce}	84.5%^{d}	77.4%^{cd}	58%^{be}
	Limited use	6.8%^{a}	8.2%^{a}	8.8%^{a}	5.3%^{a}	5.8%^{a}	5.8%^{a}
	2	7.1%^{a}	4%^{ab}	1.6%^{bcd}	0.5%^{c}	1.8%^{abc}	5.8%^{ad}
	3	19.3%^{a}	10.8%^{b}	7%^{b}	2.1%^{c}	6.2%^{bc}	13%^{ab}
	4	16.1%^{a}	9.6%^{ac}	4.3%^{b}	3%^{b}	4.4%^{bc}	4.3%^{ab}
	Extensive use	10.9%^{a}	4.7%^{bc}	3.8%^{b}	3.9%^{bc}	4.4%^{ab}	11.6%^{ac}
Tablet (Apple)	Do not use	27%^{a}	45.9%^{b}	53.6%^{bc}	60.2%^{c}	61.1%^{cd}	44.9%^{bc}
	Limited use	6.8%^{a}	7.8%^{a}	9.5%^{a}	10.2%^{a}	10.2%^{a}	10.1%^{a}
	2	4.8%^{a}	5.4%^{a}	4.1%^{a}	5.1%^{a}	1.8%^{a}	4.3%^{a}
	3	21.5%^{a}	13.2%^{b}	10.4%^{b}	8.1%^{b}	11.1%^{ab}	13%^{ab}
	4	18.3%^{a}	15.8%^{a}	8.8%^{b}	5.3%^{b}	6.2%^{ab}	7.2%^{ab}
	Extensive use	19.9%^{a}	11.8%^{b}	13.1%^{ab}	10.6%^{b}	9.7%^{b}	17.4%^{ab}
Tablet (Android)	Do not use	39.5%^{a}	56.2%^{b}	70.1%^{c}	77.1%^{c}	72.6%^{c}	49.3%^{ab}
	Limited use	8.7%^{a}	10.8%^{a}	9.7%^{a}	6.9%^{a}	9.7%^{a}	5.8%^{a}
	2	6.1%^{a}	5.6%^{a}	3.2%^{a}	2.5%^{a}	2.2%^{a}	5.8%^{a}
	3	17.7%^{a}	10.1%^{b}	6.1%^{b}	5.1%^{b}	4.9%^{b}	11.6%^{ab}
	4	11.9%^{a}	8.2%^{ab}	5%^{bc}	3.5%^{c}	5.3%^{abc}	8.7%^{abc}

Device	Usage	Lifestyle Mavens *n* = 311	Health Conscious *n* = 425	Aspirational Healthy Eaters *n* = 556	Balanced All-rounders *n* = 432	Contemplating Another Day *n* = 226	Blissfully Unconcerned *n* = 69
	Extensive use	14.8%^{a}	8.7%^{ab}	5.2%^{b}	4.2%^{bc}	4.9%^{bd}	17.4%^{a}
Tablet (Other)	Do not use	43.1%^{a}	63.5%^{b}	76.3%^{ce}	87.7%^{d}	82.7%^{cd}	60.9%^{abe}
	Limited use	7.4%^{a}	9.2%^{a}	8.6%^{a}	5.8%^{a}	6.6%^{a}	5.8%^{a}
	2	3.9%^{a}	3.3%^{a}	2.0%^{a}	1.4%^{a}	1.8%^{a}	1.4%^{a}
	3	17.0%^{a}	9.4%^{bc}	5.4%^{bc}	2.1%^{c}	4.0%^{bc}	14.5%^{ad}
	4	15.8%^{a}	5.6%^{bc}	4.1%^{bc}	1.2%^{c}	3.5%^{bc}	7.2%^{ab}
	Extensive use	11.3%^{a}	8.0%^{bc}	3.4%^{bc}	1.2%^{b}	1.3%^{b}	8.7%^{ac}
Desktop computer	Do not use	24.8%^{a}	35.5%^{b}	47.1%^{c}	57.4%^{d}	49.1%^{cd}	44.9%^{bcd}
	Limited use	7.1%^{a}	9.6%^{a}	8.1%^{a}	10.0%^{a}	11.9%^{a}	5.8%^{a}
	2	6.4%^{a}	6.6%^{a}	7.0%^{a}	4.6%^{a}	7.5%^{a}	7.2%^{a}
	3	21.5%^{a}	12.7%^{b}	12.4%^{b}	9.5%^{b}	9.3%^{b}	17.4%^{ab}
	4	18.6%^{a}	15.8%^{ab}	10.6%^{c}	4.9%^{c}	6.2%^{cd}	7.2%^{abc}
	Extensive use	20.6%^{a}	19.3%^{a}	14.4%^{a}	13.2%^{a}	15.9%^{a}	15.9%^{a}
Laptop computer	Do not use	9.6%^{a}	11.8%^{a}	12.9%^{a}	10.9%^{a}	12.8%^{a}	17.4%^{a}
	Limited use	5.1%^{ab}	3.5%^{a}	7.7%^{ab}	6.5%^{ab}	9.3%^{b}	7.2%^{ab}
	2	6.1%^{a}	5.4%^{a}	8.6%^{a}	5.8%^{a}	5.3%^{a}	5.8%^{a}
	3	19.3%^{a}	18.4%^{a}	14.7%^{a}	12.0%^{a}	15.9%^{a}	18.8%^{a}
	4	26.0%^{a}	24.7%^{a}	19.4%^{a}	20.6%^{a}	16.4%^{a}	20.3%^{a}
	Extensive use	32.2%^{a}	36.2%^{ab}	36.3%^{ab}	44.2%^{b}	40.3%^{ab}	29.0%^{ab}
Apple watch	Do not use	39.9%^{a}	59.3%^{b}	72.8%^{ce}	83.8%^{d}	79.2%^{cd}	58.0%^{abe}
	Limited use	8.0%^{a}	7.5%^{a}	7.2%^{a}	5.8%^{a}	5.3%^{a}	5.8%^{a}
	2	5.5%^{a}	3.5%^{ab}	2.3%^{ab}	1.4%^{b}	2.2%^{ab}	1.4%^{ab}
	3	15.4%^{a}	12.2%^{ab}	7.4%^{b}	2.1%^{c}	5.8%^{bcd}	13.0%^{ab}
	4	16.7%^{a}	8.0%^{b}	4.1%^{bc}	2.5%^{c}	3.5%^{bc}	7.2%^{abc}
	Extensive use	12.9%^{a}	9.2%^{ab}	5.8%^{bce}	3.5%^{c}	4.0%^{bcde}	13.0%^{ae}
Fitbit	Do not use	41.8%^{a}	54.8%^{b}	68.0%^{c}	78.2%^{d}	76.1%^{cd}	58.0%^{abc}
	Limited use	9.3%^{a}	7.5%^{a}	9.4%^{a}	6.0%^{a}	7.1%^{a}	7.2%^{a}
	2	4.8%^{a}	4.9%^{a}	3.6%^{a}	2.1%^{a}	3.1%^{a}	4.3%^{a}
	3	15.1%^{a}	11.1%^{ac}	5.6%^{b}	3.5%^{b}	5.3%^{bc}	17.4%^{a}
	4	13.2%^{a}	8.7%^{ab}	5.9%^{bc}	3.7%^{c}	4.0%^{bcd}	4.3%^{abc}
	Extensive use	14.1%^{a}	12.5%^{a}	6.8%^{b}	5.8%^{b}	4.4%^{b}	7.2%^{ab}

Device	Usage	Lifestyle Mavens *n* = 311	Health Conscious *n* = 425	Aspirational Healthy Eaters *n* = 556	Balanced All-rounders *n* = 432	Contemplating Another Day *n* = 226	Blissfully Unconcerned *n* = 69
Other wearable fitness device	Do not use	42.8%[a]	63.1%[b]	74.6%[ce]	87.0%[d]	80.5%[cd]	59.4%[abe]
	Limited use	8.0%[a]	8.7%[a]	8.5%[a]	6.3%[a]	7.1%[a]	7.2%[a]
	2	7.1%[a]	4.9%[ab]	1.8%[bc]	0.9%[c]	1.8%[bcd]	0.0%
	3	15.8%[a]	9.2%[ab]	4.9%[b]	1.4%[c]	4.4%[bcd]	15.9%[a]
	4	14.1%[a]	5.4%[b]	5.4%[b]	2.1%[b]	4.0%[b]	4.3%[ab]
	Extensive use	11.3%[a]	8.5%[ab]	4.5%[bce]	1.9%[c]	2.2%[cd]	11.6%[ae]

Supplementary Table S4: Social media platform usage by Living and Eating for Health Segments (LEHS) (n = 2019).

Platform	Usage	Lifestyle Mavens *n* = 311	Health Conscious *n* = 425	Aspirational Healthy Eaters *n* = 556	Balanced All-rounders *n* = 432	Contemplating Another Day *n* = 226	Blissfully Unconcerned *n* = 69
Facebook	Do not use	7.1%[a]	6.8%[a]	7.9%[a]	10.9%[a]	8.8%[a]	7.2%[a]
	Limited use	14.1%[a]	9.2%[a]	9.5%[a]	7.6%[a]	11.5%[a]	2.9%[a]
	2	9.6%[a]	8.9%[a]	6.8%[a]	6.9%[a]	10.2%[a]	8.7%[a]
	3	20.3%[ab]	20.7%[ab]	16.0%[a]	14.1%[a]	17.3%[ab]	33.3%[b]
	4	19.9%[ab]	24.7%[a]	18.9%[ab]	21.1%[ab]	13.7%[b]	15.9%[ab]
	Extensive use	28.6%[a]	29.6%[a]	40.8%[b]	39.1%[b]	38.5%[ab]	31.9%[ab]
YouTube	Do not use	1.6%[a]	2.6%[a]	2.5%[a]	2.5%[a]	2.2%[a]	4.3%[a]
	Limited use	6.4%[a]	4.7%[a]	5.0%[a]	6.7%[a]	5.3%[a]	1.4%[a]
	2	11.3%[a]	7.8%[a]	6.1%[a]	6.7%[a]	9.7%[a]	4.3%[a]
	3	25.1%[a]	20.5%[ab]	15.5%[b]	17.6%[ab]	20.8%[ab]	18.8%[ab]
	4	25.4%[a]	26.1%[a]	25.0%[a]	22.7%[ab]	13.3%[b]	21.7%[ab]
	Extensive use	29.9%[a]	38.1%[ab]	45.9%[b]	43.8%[bc]	48.7%[bd]	49.3%[be]
Instagram	Do not use	5.8%[a]	10.4%[ab]	12.4%[b]	15.0%[bc]	17.7%[bd]	13.0%[ab]
	Limited use	6.8%[a]	6.6%[a]	4.9%[a]	7.2%[a]	8.4%[a]	10.1%[a]
	2	8.0%[a]	8.5%[a]	6.3%[a]	5.3%[a]	8.8%[a]	10.1%[a]
	3	26.0%[a]	17.2%[b]	14.6%[b]	15.5%[b]	16.4%[ab]	26.1%[ab]
	4	23.2%[a]	23.3%[a]	18.5%[a]	16.4%[a]	19.0%[a]	20.3%[a]
	Extensive use	29.6%[a]	34.1%[ab]	43.2%[bcd]	40.3%[bcd]	29.6%[ad]	20.3%[a]
Twitter	Do not use	26.7%[a]	38.8%[b]	45.7%[b]	55.8%[c]	48.2%[bc]	39.1%[abc]
	Limited use	12.9%[a]	16.9%[a]	18.2%[a]	15.3%[a]	16.4%[a]	14.5%[a]
	2	8.4%[a]	8.2%[a]	8.5%[a]	6.5%[a]	7.1%[a]	7.2%[a]
	3	20.9%[a]	14.1%[ab]	9.4%[b]	10.0%[bc]	11.1%[bd]	18.8%[ab]
	4	18.3%[a]	13.6%[ab]	8.3%[b]	7.6%[bc]	8.4%[bd]	13.0%[ab]
	Extensive use	12.5%[a]	8.0%[ab]	9.9%[a]	4.4%[b]	8.8%[ab]	5.8%[ab]
Snapchat	Do not use	13.5%[a]	17.6%[ab]	17.4%[ab]	23.1%[b]	25.7%[bc]	29.0%[bd]
	Limited use	10.3%[a]	9.4%[a]	8.3%[a]	11.6%[a]	10.6%[a]	8.7%[a]
	2	7.7%[a]	8.2%[a]	8.8%[a]	9.5%[a]	14.2%[a]	8.7%[a]
	3	19.6%[ab]	20.5%[a]	17.3%[ab]	12.7%[b]	12.8%[ab]	21.7%[ab]
	4	23.8%[a]	20.5%[ab]	14.2%[bc]	17.1%[ab]	8.8%[c]	10.1%[abc]
	Extensive use	24.1%[a]	23.8%[a]	34.0%[b]	25.7%[ab]	27.9%[ab]	20.3%[ab]

Platform	Usage	Lifestyle Mavens *n* = 311	Health Conscious *n* = 425	Aspirational Healthy Eaters *n* = 556	Balanced All-rounders *n* = 432	Contemplating Another Day *n* = 226	Blissfully Unconcerned *n* = 69
Pinterest	Do not use	28.9%^{a}	40.2%^{b}	42.6%^{b}	46.3%^{b}	50.0%^{b}	50.7%^{b}
	Limited use	11.6%^{a}	13.9%^{ab}	20.5%^{b}	15.7%^{ab}	15.5%^{ab}	11.6%^{ab}
	2	8.7%^{a}	11.1%^{a}	9.7%^{a}	12.3%^{a}	10.2%^{a}	4.3%^{a}
	3	20.3%^{a}	14.4%^{ab}	12.1%^{b}	9.3%^{bc}	12.8%^{ab}	14.5%^{ab}
	4	19.6%^{a}	12.2%^{acd}	5.6%^{b}	9.7%^{bc}	5.3%^{bd}	7.2%^{ab}
	Extensive use	10.0%^{a}	7.8%^{a}	9.4%^{a}	6.3%^{a}	6.2%^{a}	8.7%^{a}
LinkedIn	Do not use	33.8%^{a}	48.7%^{b}	61.7%^{c}	65.7%^{c}	62.8%^{c}	56.5%^{bc}
	Limited use	12.2%^{a}	14.4%^{a}	16.2%^{a}	17.6%^{a}	14.2%^{a}	11.6%^{a}
	2	7.7%^{a}	7.8%^{a}	5.8%^{a}	5.3%^{a}	8.0%^{a}	2.9%^{a}
	3	18.6%^{a}	16.0%^{a}	7.6%^{b}	5.3%^{b}	9.7%^{ab}	14.5%^{ab}
	4	17.0%^{a}	8.2%^{b}	6.7%^{bc}	3.5%^{c}	3.1%^{bc}	4.3%^{abc}
	Extensive use	9.6%^{a}	4.5%^{ab}	2.0%^{b}	1.6%^{bc}	2.2%^{bd}	7.2%^{ab}
Tumblr	Do not use	32.8%^{a}	52.2%^{b}	52.5%^{b}	58.1%^{b}	53.1%^{b}	47.8%^{ab}
	Limited use	12.5%^{a}	12.2%^{a}	17.4%^{a}	17.6%^{a}	16.4%^{a}	5.8%^{a}
	2	9.3%^{a}	8.7%^{a}	9.2%^{a}	8.3%^{a}	8.0%^{a}	5.8%^{a}
	3	19.9%^{a}	11.3%^{bc}	10.1%^{bc}	6.0%^{b}	12.4%^{ac}	15.9%^{ac}
	4	17.0%^{a}	8.9%^{b}	5.6%^{b}	5.1%^{b}	5.8%^{ab}	5.8%^{ab}
	Extensive use	8.0%^{ab}	6.4%^{ab}	5.0%^{a}	4.4%^{a}	4.4%^{a}	14.5%^{b}
Blogspot	Do not use	39.9%^{a}	60.7%^{b}	72.8%^{c}	80.8%^{d}	76.5%^{cd}	60.9%^{bc}
	Limited use	10.9%^{a}	10.1%^{a}	13.1%^{a}	10.0%^{a}	9.7%^{a}	14.5%^{a}
	2	9.0%^{a}	8.0%^{a}	2.9%^{b}	2.8%^{b}	3.1%^{ab}	2.9%^{ab}
	3	19.0%^{a}	8.2%^{bd}	5.2%^{bc}	2.1%^{c}	6.2%^{bcd}	14.5%^{ad}
	4	12.2%^{a}	8.7%^{ac}	3.6%^{b}	2.3%^{b}	3.1%^{bc}	2.9%^{ab}
	Extensive use	8.7%^{a}	3.8%^{ab}	2.2%^{b}	1.4%^{bc}	1.3%^{bd}	2.9%^{ab}
Wordpress	Do not use	38.6%^{a}	59.8%^{b}	71.0%^{c}	80.1%^{d}	72.6%^{cd}	58.0%^{bc}
	Limited use	12.2%^{a}	11.1%^{a}	14.0%^{a}	11.1%^{a}	12.4%^{a}	17.4%^{a}
	2	9.3%^{a}	7.5%^{a}	2.3%^{b}	2.1%^{b}	5.8%^{ab}	4.3%^{ab}
	3	14.5%^{a}	8.9%^{ab}	7.2%^{b}	2.8%^{c}	5.3%^{bcd}	10.1%^{ab}
	4	15.1%^{a}	6.8%^{b}	3.1%^{bc}	1.9%^{c}	2.7%^{bc}	4.3%^{abc}
	Extensive use	9.6%^{a}	5.4%^{ab}	2.2%^{bc}	1.6%^{c}	1.3%^{bcd}	4.3%^{abc}
	Do not use	38.9%^{a}	58.6%^{b}	71.8%^{cd}	78.7%^{c}	76.1%^{cd}	60.9%^{bd}

Platform	Usage	Lifestyle Mavens *n* = 311	Health Conscious *n* = 425	Aspirational Healthy Eaters *n* = 556	Balanced All-rounders *n* = 432	Contemplating Another Day *n* = 226	Blissfully Unconcerned *n* = 69
Other blog platforms	Limited use	11.9%^{a}	12.0%^{a}	12.4%^{a}	11.1%^{a}	10.6%^{a}	11.6%^{a}
	2	7.1%^{a}	6.8%^{a}	2.5%^{b}	1.9%^{b}	2.2%^{ab}	5.8%^{ab}
	3	17.0%^{a}	8.0%^{bc}	6.8%^{bc}	3.5%^{b}	4.9%^{bc}	13.0%^{ac}
	4	13.2%^{a}	9.4%^{bc}	4.0%^{b}	2.3%^{b}	4.4%^{bc}	5.8%^{ab}
	Extensive use	10.9%^{a}	4.9%^{b}	2.3%^{b}	2.1%^{b}	1.8%^{b}	1.4%^{ab}
Spotify	Do not use	17.4%^{a}	20.2%^{ab}	21.6%^{ab}	26.6%^{b}	30.5%^{bc}	34.8%^{bd}
	Limited use	7.4%^{ab}	4.9%^{ab}	8.6%^{ab}	10.6%^{b}	10.2%^{ab}	10.1%^{ab}
	2	8.4%^{ab}	9.2%^{ab}	5.6%^{ab}	4.2%^{b}	5.8%^{ab}	7.2%^{ab}
	3	21.5%^{a}	15.8%^{bd}	13.3%^{bd}	10.0%^{bc}	14.2%^{ab}	23.2%^{ad}
	4	20.6%^{a}	21.9%^{ab}	15.6%^{ab}	17.6%^{ab}	11.1%^{ba}	8.7%^{ab}
	Extensive use	24.1%^{a}	27.8%^{b}	35.3%^{b}	30.8%^{ab}	28.3%^{ab}	14.5%^{a}
Soundcloud	Do not use	31.2%^{a}	47.8%^{b}	55.2%^{bc}	62.3%^{c}	65.0%^{cd}	47.8%^{abc}
	Limited use	10.3%^{a}	12.2%^{a}	15.3%^{a}	15.5%^{a}	10.2%^{a}	14.5%^{a}
	2	10.3%^{a}	7.8%^{a}	7.0%^{a}	7.4%^{a}	5.3%^{a}	5.8%^{a}
	3	20.3%^{a}	15.8%^{ab}	10.4%^{b}	4.9%^{c}	11.5%^{ab}	18.8%^{ab}
	4	15.4%^{a}	8.5%^{b}	5.0%^{b}	5.8%^{b}	3.5%^{b}	4.3%^{ab}
	Extensive use	11.9%^{a}	7.8%^{ab}	6.8%^{ab}	3.9%^{b}	4.4%^{bc}	7.2%^{ab}
Reddit	Do not use	33.4%^{a}	47.8%^{b}	51.3%^{b}	55.8%^{b}	49.1%^{b}	47.8%^{ab}
	Limited use	11.6%^{a}	12.5%^{a}	15.6%^{a}	14.1%^{a}	15.9%^{a}	13.0%^{a}
	2	9.3%^{a}	8.2%^{a}	7.0%^{a}	10.2%^{a}	6.2%^{a}	5.8%^{a}
	3	18.6%^{a}	12.5%^{ab}	10.6%^{b}	7.4%^{bc}	13.7%^{ab}	17.4%^{ab}
	4	15.8%^{a}	12.0%^{ab}	7.2%^{bc}	5.8%^{c}	8.4%^{abc}	8.7%^{abc}
	Extensive use	10.3%^{a}	6.8%^{a}	8.1%^{a}	6.5%^{a}	6.6%^{a}	5.8%^{a}
Other forums/discussion boards	Do not use	37.0%^{a}	54.6%^{b}	63.3%^{bc}	71.3%^{c}	62.8%^{bc}	56.5%^{bc}
	Limited use	10.3%^{a}	13.6%^{a}	14.2%^{a}	12.5%^{a}	13.7%^{a}	15.9%^{a}
	2	8.0%^{a}	6.6%^{a}	4.5%^{a}	4.9%^{a}	4.4%^{a}	4.3%^{a}
	3	20.9%^{a}	11.8%^{b}	8.8%^{bc}	5.6%^{c}	8.8%^{bc}	13.0%^{abc}
	4	12.2%^{a}	8.2%^{ac}	6.8%^{ab}	3.0%^{b}	3.5%^{bc}	5.8%^{ab}
	Extensive use	10.9%^{a}	4.9%^{bc}	2.2%^{b}	2.3%^{bc}	6.6%^{ac}	2.9%^{ab}

Supplementary Table S5: Online behaviours with social media content by Living and Eating for Health Segments (LEHS) (n = 2019).

Online behaviour		Lifestyle Mavens n = 311	Health Conscious n = 425	Aspirational Healthy Eaters n = 556	Balanced All-rounders n = 432	Contemplating Another Day n = 226	Blissfully Unconcerned n = 69
Sharing opinions	Not really applicable	11.3%[a]	22.4%[b]	27.5%[bd]	37.5%[c]	35.4%[cd]	31.9%[bc]
	2	14.8%[a]	15.5%[a]	20.5%[a]	19.9%[a]	19.5%[a]	13.0%[a]
	3	43.4%[a]	35.1%[ab]	28.8%[b]	18.8%[c]	24.8%[bcd]	42.0%[ab]
	4	19.0%[a]	18.1%[a]	14.6%[a]	15.5%[a]	11.1%[a]	7.2%[a]
	Highly applicable	11.3%[a]	8.9%[a]	8.6%[a]	8.3%[a]	9.3%[a]	5.8%[a]
Creating content	Not really applicable	11.3%[a]	20.0%[b]	25.2%[bc]	32.4%[cd]	35.8%[d]	31.9%[bcd]
	2	20.6%[a]	19.3%[a]	21.6%[a]	15.5%[a]	17.7%[a]	15.9%[a]
	3	38.3%[a]	30.1%[ab]	28.1%[b]	27.1%[bc]	22.6%[bd]	37.7%[ab]
	4	18.6%[a]	20.0%[a]	14.9%[ab]	16.0%[ab]	13.3%[ab]	4.3%[b]
	Highly applicable	10.6%[a]	10.6%[a]	10.3%[a]	9.0%[a]	10.6%[a]	10.1%[a]
Following content	Not really applicable	4.2%[a]	7.3%[a]	5.2%[a]	9.0%[a]	9.7%[a]	13.0%[a]
	2	12.5%[a]	8.9%[a]	10.4%[a]	8.6%[a]	9.3%[a]	8.7%[a]
	3	37.9%[a]	34.1%[abc]	32.4%[abc]	27.3%[b]	27.9%[ab]	49.3%[c]
	4	25.7%[a]	30.6%[a]	29.0%[a]	27.3%[a]	29.6%[a]	14.5%[a]
	Highly applicable	18.6%[a]	19.1%[a]	23.0%[ab]	27.8%[b]	23.5%[ab]	14.5%[ab]
Listening to content	Not really applicable	4.8%[a]	6.8%[ab]	7.2%[ab]	9.0%[ab]	13.7%[b]	8.7%[ab]
	2	11.6%[a]	8.7%[a]	8.1%[a]	6.5%[a]	9.3%[a]	11.6%[a]
	3	35.7%[a]	29.9%[ab]	30.8%[ab]	28.9%[ab]	21.7%[b]	42.0%[a]
	4	27.7%[a]	30.6%[a]	27.2%[a]	25.5%[a]	25.7%[a]	14.5%[a]
	Highly applicable	19.3%[a]	23.8%[ab]	26.8%[ab]	30.1%[b]	29.2%[b]	23.2%[ab]
Reviewing content	Not really applicable	7.4%[a]	15.5%[a]	17.3%[b]	25.2%[c]	30.5%[c]	20.3%[bc]
	2	11.6%[a]	12.9%[ab]	20.0%[b]	20.1%[bc]	19.0%[ab]	18.8%[ab]
	3	39.5%[a]	34.6%[ab]	32.6%[ab]	25.7%[b]	28.3%[ab]	34.8%[ab]
	4	26.7%[a]	24.2%[ab]	17.1%[bc]	18.3%[abc]	10.6%[c]	18.8%[abc]
	Highly applicable	13.8%[a]	12.7%[a]	13.1%[a]	10.6%[a]	11.1%[a]	7.2%[a]
Sharing content	Not really applicable	8.7%[a]	13.9%[ab]	18.5%[bd]	18.3%[bcd]	23.0%[d]	23.2%[bde]
	2	15.8%[a]	17.6%[a]	15.8%[a]	21.1%[a]	14.2%[a]	10.1%[a]
	3	36.3%[a]	34.6%[a]	29.1%[ab]	25.2%[b]	28.3%[ab]	39.1%[ab]
	4	25.4%[a]	22.4%[a]	21.0%[a]	19.4%[a]	19.0%[a]	15.9%[a]
	Highly applicable	12.9%[a]	11.5%[a]	15.5%[a]	16.0%[a]	15.0%[a]	11.6%[a]
	Not really applicable	3.5%[a]	7.5%[ab]	5.9%[ab]	6.3%[ab]	11.5%[b]	14.5%[bc]

**Online behaviour**		**Lifestyle Mavens** ***n*** **= 311**	**Health Conscious** ***n*** **= 425**	**Aspirational Healthy Eaters** ***n*** **= 556**	**Balanced All-rounders** ***n*** **= 432**	**Contemplating Another Day** ***n*** **= 226**	**Blissfully Unconcerned** ***n*** **= 69**
Researching content	2	13.5%[a]	9.9%[a]	9.2%[a]	8.6%[a]	13.3%[a]	14.5%[a]
	3	33.4%[a]	32.0%[a]	30.4%[a]	21.5%[b]	26.5%[ab]	36.2%[ab]
	4	30.2%[a]	26.1%[a]	25.9%[a]	29.6%[a]	26.5%[a]	17.4%[a]
	Highly applicable	18.3%[a]	24.5%[ab]	28.6%[bcd]	34.0%[c]	21.7%[ad]	17.4%[abc]
Entertainment online	Not really applicable	4.5%[a]	4.9%[a]	4.1%[a]	4.9%[a]	4.0%[a]	7.2%[a]
	2	12.9%[a]	8.0%[ab]	7.7%[ab]	4.4%[b]	5.3%[bc]	5.8%[ab]
	3	37.0%[a]	36.0%[a]	21.8%[bc]	19.7%[b]	20.4%[bc]	36.2%[ac]
	4	27.7%[a]	23.5%[a]	25.9%[a]	28.0%[a]	21.7%[a]	20.3%[a]
	Highly applicable	17.0%[a]	27.5%[b]	40.3%[c]	43.1%[c]	48.2%[c]	30.4%[abc]
SNS for work	Not really applicable	5.1%[a]	11.5%[b]	16.7%[bc]	22.0%[c]	22.6%[cd]	24.6%[ce]
	2	9.6%[ab]	8.9%[a]	17.1%[bc]	14.1%[abc]	18.6%[c]	18.8%[abc]
	3	39.9%[a]	32.2%[ab]	33.1%[ab]	25.5%[b]	31.4%[ab]	33.3%[ab]
	4	28.6%[a]	28.2%[a]	18.0%[b]	18.8%[b]	14.6%[b]	13.0%[ab]
	Highly applicable	15.4%[a]	19.1%[a]	15.1%[a]	19.7%[a]	12.4%[a]	10.1%[a]
